# Microparticles expressing myeloperoxidase as potential biomarkers in anti-neutrophil cytoplasmic antibody (ANCA)-associated vasculitides (AAV)

**DOI:** 10.1007/s00109-020-01955-2

**Published:** 2020-07-30

**Authors:** M. Manojlovic, A. Juto, A. Jonasdottir, J. Colic, J. Vojinovic, A. Nordin, A. Bruchfeld, I. Gunnarsson, F. Mobarrez, A. Antovic

**Affiliations:** 1 Department of Pediatrics, Medical Faculty, University of Nis, Nis, Serbia; 2 Department of Medicine, Division of Rheumatology Karolinska Institutet and Rheumatology, Karolinska University Hospital, Stockholm, Sweden; 3 Renal medicine, CLINTEC, Karolinska University Hospital and Karolinska Institutet, Solna, Stockholm, Sweden; 4 Department of Rheumatology, Institute of Rheumatology, Belgrade, Serbia; 5 Department of Medical Sciences, Uppsala University, Uppsala, Sweden

**Keywords:** Anti-neutrophil cytoplasmic antibody (ANCA)-associated vasculitis, Biomarkers, Microparticles

## Abstract

**Abstract:**

To investigate presence of circulating myeloperoxidase-positive microparticles (MPO^+^MPs) in relation to disease activity in patients with anti-neutrophil cytoplasmic antibody (ANCA)-associated vasculitis (AAV). Forty-six patients with AAV and 23 age- and sex-matched healthy controls were included. Vasculitis disease activity was assessed using the Birmingham Vasculitis Activity Score (BVAS). MPs were analyzed in citrate plasma by flow cytometry and phenotyped based on MPO expression and co-expression of pentraxin-3 (PTX3), high mobility group box 1 protein (HMGB1), and tumor necrosis factor-like weak inducer of apoptosis (TWEAK). Serum levels of PTX3, sTWEAK, and HMGB1 were also determined. Twenty-three patients had active vasculitis (BVAS ≥ 1). Concentrations of MPO^+^MPs expressing PTX3, HMGB1, and TWEAK were significantly higher in patients compared to healthy controls (*p* < 0.001, *p* < 0.01, *p* < 0.001, respectively), while concentrations of PTX3^+^ and HMGB1^+^MPO^+^MPs were significantly higher in active AAV compared to patients in remission. MPO^+^MPs expressing either PTX3 or HMGB1 were associated with BVAS (*r* = 0.5, *p* < 0.001; *r* = 0.3, *p* = 0.04, respectively). Significantly higher serum PTX3 levels were found in active- than in inactive AAV (*p* < 0.001), correlating strongly with BVAS (*r* = 0.7, *p* < 0.001). Serum levels of sTWEAK and HMGB1 did not differ between patients and controls. Concentration of MPO^+^MPs is increased in plasma from AAV patients compared to healthy individuals. PTX3 in serum as well as PTX3 and HMGB1 expressed on MPO^+^MPs were associated with disease activity in the investigated patients.

**Key messages:**

- Myeloperoxidase-positive microparticles (MPO^+^MPs) are increased in plasma from patients with ANCA-associated vasculitis.
- Concentrations of MPO^+^MPs expressing PTX3, HMGB1, and TWEAK were significantly higher in patients compared to healthy controls.
- MPO^+^MPs expressing PTX3 and HMGB1 are associated with disease activity in ANCA-associated vasculitis.

**Electronic supplementary material:**

The online version of this article (10.1007/s00109-020-01955-2) contains supplementary material, which is available to authorized users.

## Introduction

Antineutrophil cytoplasmic antibodies (ANCAs) are serologic hallmarks of ANCA-associated vasculitis (AAV), a group of multisystem disorders characterized by pauci-immune necrotizing vasculitis affecting small- to medium-sized blood vessels. These conditions are associated with an increased risk of irreversible organ damage, especially in the kidneys, lungs and central nervous system, as well as with an increased mortality risk. ANCAs are predominantly IgG autoantibodies directed against neutrophil cytoplasmic components, in particular proteinase 3 (PR3) and myeloperoxidase (MPO) [1].

There is a growing body of evidence that ANCA-induced neutrophil activation plays an essential role in the pathogenesis of AAV [1–3]. Priming, degranulation, and release of neutrophil extracellular traps (NETs) occurs, whereby neutrophils undergo apoptosis and necrosis [1]. Complement activation, especially via the alternative pathway, acts as positive feedback amplification of neutrophil activation, resulting in the aggressive necrotizing inflammation in ANCA-associated diseases [4].

During activation and apoptosis, neutrophils release microparticles (MPs), also known as extracellular vesicles. MPs are submicron membranous vesicles, which could exert pro-inflammatory and pro-coagulant stimuli in a course of vasculitis [5]. It has been shown that neutrophil-derived MPs contain active myeloperoxidase (MPO), an enzyme responsible for activation of endothelial cells, injury, and development of vascular lesions in AAV [6–8]. We have recently demonstrated increased levels of MPs expressing MPO and complement components C3a and C5a in patients with AAV, postulating that a majority of MPs are of neutrophil origin, although monocytes also have been shown to express MPO in lower concentrations [9].

There are several other potential molecules of importance for the pathogenesis of AAV, for instance pentraxin 3 (PTX3), an acute phase reactant, which is stored together with MPO and PR3 in neutrophil granules and released upon respiratory burst. PTX3 has been detected on NETs reflecting neutrophil reactivity in AAV [10]. Furthermore, high-mobility group box 1 (HMGB-1) protein, an important proinflammatory mediator actively secreted from monocytes and macrophages and passively released from necrotic cells, has been implicated in the pathogenesis of AAV, considering the presence of these nuclear components in MPs during cell activation and apoptosis [11]. HMGB1 has also been shown to be increased in serum of active AAV patients with kidney involvement and expressed in renal tissue [12]. HMGB1 contributes to priming neutrophils, increased translocation of ANCA antigens to cell membranes and thereby adds to antigen-antibody interactions [13]. HMGB1 can also potentiate ANCA-induced NETs formation [14]. However, it is unknown whether HMGB1 exits the cell in MPs or is released in a free form and thereafter binds to these particles [11]. Additionally, investigation of soluble tumor necrosis factor-like weak inducer of apoptosis (sTWEAK) in the pathogenesis of AAV has been shown to be of interest, knowing that sTWEAK is a potential biomarker of adverse outcomes in chronic kidney disease (CKD) [15].

The aim of this study was to assess the expression of PTX3, HMGB1, and TWEAK as candidate biomarkers of inflammatory process on MPO^+^MPs, during active disease and in remission, compared to healthy subjects.

## Patients and methods

We have performed a cross-sectional study including a group of 46 ANCA-positive patients with AAV, either with granulomatosis with polyangiitis or microscopic polyangiitis. The patients were recruited from the Departments of Nephrology and Rheumatology at Karolinska University Hospital. The assessment of vasculitis disease activity was performed using the Birmingham Vasculitis Activity Score (BVAS, version 2003), according the recommendations by the European League Against Rheumatism [16]. Half of the patients (*n* = 23) were included in the active phase of the disease. All but one patients were included after the first episode of active AAV (median disease duration 2 days (0–22 days)). The patient with relapse had a disease duration of 6 months (285 days). None of the included patients was tested repeatedly in active disease and remission. Patients with a BVAS of 0 were considered to be in remission. The inactive patients had a median disease duration of 5.3 years (range 1.2–12 years).

Renal involvement was defined as pathological changes on a recent renal biopsy (seen in 14 patients with active disease, renal biopsy performed in 13 cases, and/or by the presence of significant haematuria and/or elevated creatinine values).

Control samples were obtained from 23 healthy age and gender-matched subjects. The local ethics committee approved the study protocol, and informed consent for publication of study results was obtained from each subject.

### Blood sampling

Peripheral venous blood was collected into Vacutainer tubes (Becton Dickinson) containing trisodium citrate (0.129 mol/L, pH 7.4) (1 part trisodium citrate and 9 parts blood). Serum, respectively platelet poor plasma (PPP) was obtained within 60 min of sampling by centrifugation at 2000*g* for 20 min at room temperature, then divided into aliquots and stored frozen at − 70 °C.

### Detection of microparticles using flow cytometry

PPP was thawed in a water bath at 37 °C for approximately 5 min, followed by centrifugation of samples at 2000*g* for 20 min at room temperature, in order to remove any cells or debris that may interfere with the analysis. The supernatant was centrifuged again at 13,000*g* for 2 min. Twenty microliters of the supernatant was incubated in dark for 20 min with 5 μl of monoclonal antibodies, anti-MPO-PE (Beckman Coulter, Brea, CA, USA) together with antibodies for pentraxin 3-Dylight 755 (anti-pentraxin 3, Abcam, Cambridge, UK), HMGB1-Dylight 488 (R&D Systems, MN, USA), and TWEAK-Dylight 633 (anti-TWEAK, LSBio. Inc., Seattle, WA, USA). After incubation, samples were fixed prior to analysis (Cellfix, BD, NJ, USA). MPs were measured by flow cytometry on a Beckman Gallios instrument (Beckman coulter, Brea, CA, USA) with the threshold set to forward scatter. The MP gate was determined using Megamix plus beads (0.3–0.9 μm, BioCytex, Marseille). MPO^+^MPs were defined as particles < 0.9 μm in size and positive for anti-MPO PE. Conjugate isotype-matched immunoglobulins with no reactivity against human antigens were used as negative controls (IgG PE, IgG Dylight 633, Dylight 488, and Dylight 755, Abcam, Cambridge, UK). Results are presented as MPs/ul plasma, processed from the 20 μl supernatant obtained after centrifugation. The intra- and inter-assay coefficients of variation for MPO^+^MPs measurement were less than 9%, respectively.

### Serological markers

PR3- and MPO ANCA-titers were detected by the standard enzyme-linked immunosorbent assay method multiplex (BIO-RAD, BioPlex TM 2200) according to clinical routine at Karolinska University Hospital.

Serum levels of PTX3 were analyzed using a commercially available ELISA kit from R&D Systems Europe Ltd. (Abington, UK). Soluble TWEAK levels in serum were determined using Human TWEAK ELISA kit (Thermo Scientific, USA). Commercial Tecan HMGB1 ELISA Kit (Fisher Scientific, USA) was used to assess HMGB-1 levels in serum.

Routine laboratory analyses were carried out using standard methods at the Karolinska University Hospital, including C-reactive protein (CRP), erythrocyte sedimentation rate (ESR), and plasma creatinine levels. The Lund Malmö equation (LM Revised) was used to estimate creatinine clearance (estimated glomerular filtration rate, eGFR) [17].

### Statistical analysis

Data were analyzed using GraphPad Prism, version 4 (GraphPad Software, San Diego, CA, USA). Descriptive statistics were used for presentation of patient characteristics. For continuous variables, means and standard deviations or medians with ranges were used, whereas categorical variables were presented as percentages. Distribution of the data was checked by the Shapiro–Wilk test. Independent samples *t* tests (parametric) and Mann–Whitney *U* tests (non-parametric) were used to assess the difference in estimated variables between groups. For comparison of more than two groups of individuals, one-way ANOVA was used. To evaluate the prognostic value of investigated parameters in predicting disease activity, receiver–operator characteristic (ROC) curve analysis was performed. ROC curves are presented with respective area under the curve (AUC) and 95% confidence intervals (CI). A *p* value < 0.05 was regarded as statistically significant. Correlation between variables was examined using Pearson and Spearman correlation analysis, depending on data type and distribution.

## Results

### General patient data

Detailed characteristics of the patients and controls, including age, sex, diagnosis, ANCA antibody type, disease activity score (BVAS), and renal function, are described in Table 1. The same cohort was also a part of the recently published study [9]. There was no difference between active and inactive patients with regard to these characteristics although creatinine levels were significantly increased and eGFR levels significantly decreased in both groups of AAV-patients compared to controls, as shown in Table 1.

**Table 1:** Patients and controls general features

	Total AAV	Healthy controls	*p*	Active AAV	Inactive AAV	*p*^1^
Patient characteristics						
Subject number	46	23		23	23	
Gender (M/F)	25/21	12/11	ns	13/10	12/11	ns
Age at sampling (mean ± SD)	62.5 ± 13.3	66.0 ± 9.5	ns	61.3 ± 14.0	63.8 ± 12.8	ns
Disease characteristics						
BVAS*	1.5 (0–31)	/		14.0 ± 8.1	0	
MPA (%)	23 (50%)	/		13 (56.5)	10 (43.5)	
GPA (%)	23 (50%)	/		10 (43.5)	13 (56.5)	
MPO-ANCA positivity ever (%)	26 (54.1)^ǂ^	/		13 (54.2)	13 (54.2)	ns
PR3-ANCA positivity ever (%)	22 (45.9)^ǂ^	/		11 (45.8)	11 (45.8)	ns
Serum creatinine (μmol/l)	125.2 ± 73.4	75.5 ± 12.4	< 0.001	145.4 ± 94.2^a^	105.0 ± 35.9^b^	0.39
eGFR (ml/min/1.73m^2^)	62.8 ± 28.4	85.3 ± 16.2	< 0.001	60.7 ± 32.9^a^	64.8 ± 23.6^b^	ns
ESR (mm/h)	28.0 ± 23.4	/		37.6 ± 28.3	19.9 ± 4.4	0.03
CRP (mg/l)	6.0 (0–71.0)			10.0 (0–71.0)	2.0 (0–17.0)	0.001
Treatment at sampling						
Prednisolone dose (mg/day, median and range)	10.0 (0–75.0)	/		30.0 (0–75.0)	5.0 (0–20.0)	0.002
Methotrexate *n* (%)	7 (15.2)			3 (13.0)	4 (17.4)	ns
Azathioprine *n* (%)	7 (15.2)			1 (4.3)	6 (26.1)	0.10
Mycophenolate mofetil *n* (%)	6 (13)			5 (21.7)	1 (4.3)	0.19
Cyclophosphamide *n* (%)	5 (10.9)			5 (21.7)	0	0.05

*Birmingham Vasculitis Activity Score (BVAS) ≥ 1 is defined as active disease; ^ǂ^Two patients were double positive for PR3-ANCA and MPO-ANCA, *p* value calculated between the whole AAV group and controls, *p*^1^ value calculated between active and inactive AAV-patients, ^a^vs control group (creatinine *p* = 0.001, eGFR: *p* = 0.006), ^b^vs control group (creatinine *p* = 0.002, eGFR: *p* = 0.001). *MPO*, myeloperoxidase; *eGFR*, estimated glomerular filtration rate; *ESR*, erythrocyte sedimentation rate; *CRP*, C reactive protein; *GPA*, granulomatosis with polyangiitis; *MPA*, microscopic polyangiitis

#### Disease activity and laboratory results

Twenty-three patients had active disease defined as a BVAS > 0 (mean BVAS 13.9 ± 7.8). Table 1 also demonstrates clinical and laboratory variables of active, respectively inactive AAV patients. Serum ESR and CRP levels were significantly higher in the active AAV group (*p* = 0.03 and *p* = 0.001, respectively). Data regarding treatments in each group patients is also shown in Table 1 The mean prednisolone dose was significantly higher in active AAV patients compared to inactive (*p* = 0.002).

### Expression of different biomarkers on myeloperoxidase positive microparticles

**Fig. 1:**
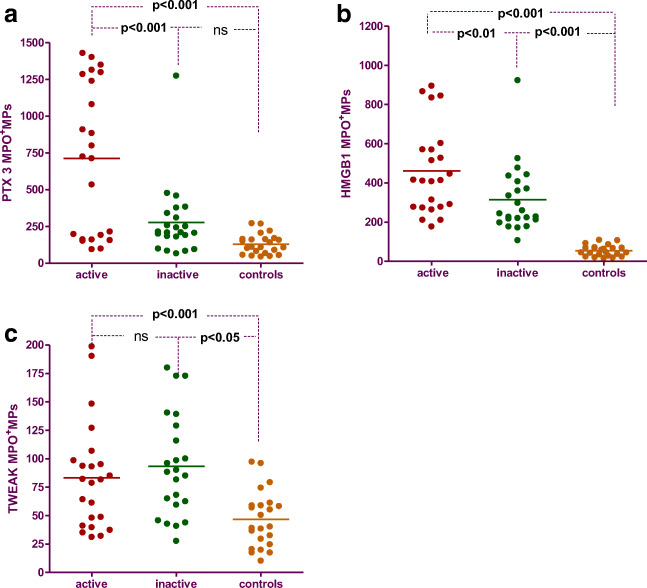
Concentration of MPO + MPs expressing PTX3 (**a**), HMGB1 (**b**), and sTWAEK (**c**) in patients with active AAV, inactive AAV, and controls

The concentrations of MPO^+^MPs expressing PTX3, HMGB1, and TWEAK were significantly higher in AAV patients compared to controls (Table 2) as well as in active compared to inactive patients except for TWEAK (Fig. 1a–c). Serum levels of PTX3 differed significantly between AAV patients and controls as well as between active AAV patients and those in remission (Table 2). No difference in serum levels of sTWEAK and HMGB1 was found between patients and controls.

**Table 2:** Concentration of MPO^+^MPs expressing PTX3, HMGB1, and TWEAK in plasma of patients with AAV and controls, as well as serum levels of these biomarkers of patients with AAV and controls

Variables	Total AAV *n* = 46	Controls *n* = 23	*p*^†^	Active AAV *n* = 23	Inactive AAV *n* = 23	*p*^‡^
Biomarkers expressed on MPO^+^MPs						
MPO	190.8 ± 28.4	46.7 ± 23.0	< 0.001	225.7 ± 50.1	154.4 ± 24.1	0.72
PTX3	245.8 (65.4–1429.0)	129.7 ± 15.6	< 0.001	724.2 (94.0–1429.0)	208.8 (65.4–1274.0)	0.001
HMGB1	324.7 (106.8–923.4)	53.6 ± 27.7	< 0.001	461.4 ± 223.5	243.6 (106.8–923.4)	0.006
TWEAK	83.5 (27.6–198.6)	46.6 ± 24.6	< 0.001	83.3 ± 47.1	93.2 ± 44.5	0.45
Serum markers						
s-PTX3 (ng/ml)	1.45 (0.4–25.7)	0.75 (0.39–2.35)	< 0.05	2.3 (0.4–25.7)	1.0 (0.5–3.4)	0.001
s-sTWEAK (pg/ml)	1340 (637–13.600)	1350 (951–3240)	0.38	1340 (976–3330)	1220 (637–13.600)	0.09
s-HMGB1 (ng/ml)	3.6 (0.9–24.2)	4.4 (1.5–15.2)	0.49	4.3 (0.9–24.2)	3.3 (1.1–20.1)	0.34

Data is presented as mean ± SD or median (range) depending on data distribution

*MPO* myeloperoxidase; *PTX3* pentraxin3; *HMGB1* high-mobility group box protein 1; *sTWEAK* soluble tumor necrosis factor-like weak inducer of apoptosis

^†^Total AVV vs healthy controls, Mann-Whitney test

^‡^Active vasculitis vs vasculitis in remission, Mann-Whitney test

‖- Mean±SD, Mann-Whitney test: (*p* < 0,01)

There was no correlation between age at the time of blood sampling, ESR, CRP, renal function (assessed by creatinine or eGFR), and the levels of the investigated markers on MPO^+^MPs or serum levels of those markers in the whole group of patients.

Fourteen patients with active AAV (60%) presented with renal flare at the time of inclusion in the study. The levels of investigated markers on MPs and in serum did not differ between patients with renal flares compared to non-renal active AAV.

Significant correlations between the disease activity measured by BVAS and the levels of PTX3 and HMGB1 expressed on MPO^+^MPs (*r* = 0.56, *p* < 0.001; *r* = 0.36, *p* = 0.01, respectively) were however observed. Regarding the serum markers, only PTX3 was strongly correlated to BVAS (*r* = 0.69, *p* < 0.001), while there was no significant correlation between other serum markers and disease activity reflected by BVAS.

There was a correlation between serum levels of PTX3 and PTX3^+^MPO^+^MPs (*r* = 0.3, *p* < 0.05), while correlations between serum sTWEAK levels and MPO^+^MPs expressing TWEAK or between HMGB1 levels in serum and HMGB1^+^MPO^+^MPs were not observed.

ROC analysis was used to assess the validity of PTX3 serum concentration and levels of MPO + MPs expressing either PTX3 or HMGB1 in identifying active AAV patients. Areas under the curves (AUCs) (95% CI) were 0.8 (0.7–0.9), 0.7 (0.6–0.9), and 0.7 (0.5–0.9), respectively. The results are presented in Supplementary file, Figure 1.

There were no differences in the MPO^+^MPs expressing PTX3, HMGB1 or TWEAK, or serum levels of these proteins comparing MPO- and PR3-ANCA-positive patients.

In the group of active patients, 5 out of 23 patients were sampled before onset of corticosteroids treatment. The concentrations of PTX3, HMGB1, or TWEAK expressed on MPO^+^MPs and in serum in these patients were not different as compared with the rest of the patients in the active group. There was no correlation between the prednisolone-dose at the time of sampling and levels of investigated markers expressed on MPO^+^MPs in the group of active patients.

In the group of inactive patients, 8 out of 23 patients were not treated with prednisolone at the time of blood sampling. The levels of investigated parameters did not differ between patients with ongoing prednisolone treatment and those without treatment.

## Discussion

In the present study, we have demonstrated significantly increased concentrations of MPO^+^MPs expressing inflammatory biomarkers PTX3, HMGB1, and TWEAK in plasma samples from AAV patients compared to healthy controls, while concentrations of MPO^+^MPs expressing PTX3 and HMGB1 were significantly higher in active AAV compared to patients in remission. Moreover, we have observed correlations between the disease activity measured by BVAS and levels of PTX3^+^- and HMGB1^+^MPO^+^MPs. Hence, by presenting these inflammatory biomarkers, MPO^+^MPs may contribute to endothelial activation and amplification of vascular inflammation in the course of AAV. Our group has recently elucidated the potential role of MPO^+^MPs expressing complement components C3a and C5a in AAV, particularly in patients with renal flare [9]. However, it seems from the present results that presence of PTX3, HMGB1, and TWEAK on MPO^+^MPs was not specifically associated to renal involvement in active AAV patients.

This was a pilot study, designed to investigate candidate biomarkers of disease activity specific for AAV including HMGB1, PTX3, and TWEAK. Previously, these biomarkers have been recognized as potential players in the pathogenesis of AAV. The novelty of this study is simultaneous evaluation of these biomarkers expressed on MPs originated mostly from neutrophils. According to our results, determination of HMGB1+ and PTX3+ MPO + MPs is associated with disease activity in AAV. Due to the pilot design of the study and relatively low number of investigated patients, we were not able to demonstrate that simultaneous measurement of more than one biomarker would improve the correlation with disease activity. Still, we believe that the results presented in our study add to the knowledge in the biomarker field in AAV.

Investigation of MPs in AAV started back in 2004, when Brogan et al. [18] demonstrated increased circulating levels of MPs (mainly of endothelial cell origin, but also platelet derived) that correlated with disease activity in children with active systemic vasculitis. Subsequently, Erdbruegger et al. confirmed this observation in adults with active vasculitis [19]. Later, the focus has changed to neutrophil-derived MPs (NMPs), and several studies have demonstrated the putative role of NMPs in activation of endothelial cells and vascular damage in AAV [6–8]. These studies provide evidence that human NMPs contain active MPO, suggesting that they may activate endothelial cells via reactive oxygen species (ROS)-dependent mechanism [7].

Together with MPO, PTX3 is released from neutrophil granules upon inflammatory burst. Consequently, PTX3 could be associated with the parent cell via NETs [20], and expression of PTX3 on MPs may in turn reflect neutrophil reactivity in AAV. A correlation between levels of PTX3 and disease activity assessed by BVAS, independently of CRP and creatinine, was previously exhibited [21]. Interestingly, almost 40% of AAV patients have been shown to harbor anti-PTX3 antibodies [22]. Serum levels of these antibodies were increased in AAV patients compared to healthy subjects and found to be higher in active disease. Moreover, in longitudinal analysis, anti-PTX3 antibodies reactivity decreased significantly after remission, suggesting a possible involvement of these antibodies in the vasculitis pathophysiology, together with other ANCA antibodies. Our results could further point towards the role of this acute phase protein in the inflammatory process of AAV, since both the increased expression of PTX3 on MPO^+^ MPs and serum levels correlated with disease activity in the investigated patients.

MPO^+^MPs expressing TWEAK were significantly increased in AAV patients compared to healthy controls. However, neither augmentation of MPO^+^MPs expressing TWEAK nor serum levels of sTWEAK were correlated to disease activity measured by BVAS. Expression of sTWEAK has been observed in other autoimmune diseases, such as lupus nephritis [23], but to the best of our knowledge, its potential role in AAV has not been elucidated. A majority of the investigated patients with active AAV (60%) in our study had ongoing renal involvement; however, TWEAK levels expressed on MPO^+^MPs and in serum did not differ in this subgroup of patients compared to other active AAV patients. Furthermore, there was no correlation found between the levels of TWEAK-positive MPO^+^MPs and renal function estimated by creatinine levels or eGFR in the investigated group of AAV patients. Therefore, we were not able to ascertain the role of sTWEAK in AAV patients with renal involvement in our cohort.

Elevated serum levels of HMGB1 in AAV patients with renal involvement measured by Western Blot compared to patients in remission were demonstrated previously by Bruchfeld A et al. [11]. In another AAV cohort [24], plasma levels of HMGB1 correlated with BVAS, serum creatinine, and eGFR, suggesting that circulating HMGB1 might reflect the disease activity and renal involvement of AAV. The same authors [25] reported increased urinary HMGB1 levels in active AAV patients, which correlated with disease activity. On the contrary, other studies found no difference regarding serum HMGB1 levels among AAV disease subsets and healthy controls [26]. However, Urbonaviciute et al*.* have previously found a discrepancy between Western blot and ELISA results, suggesting that serum/plasma components bind to HMGB1 and interfere with its detection by ELISA systems [27]. As reviewed by Harris et al. [28] and Pitzesky [11], HMGB1 is present in a nuclear material from both activated and apoptotic cells. Thus, HMGB1 as a component of MPO^+^MPs microparticles may have dual origin during the course of the inflammatory and necrotic processes in AAV. The highest concentration of HMGB1 bearing MPO^+^MPs was found in samples from AAV-patients with active disease further pointing to a putative role of HMGB1 in AAV pathophysiology. Furthermore, posttranslational modifications, oxidation/reduction, and interaction with other bioactive molecules might affect the bioactivity of HMGB1 [29] which is not captured by measuring the total concentration of the molecule in the blood [11, 29]. Hence, the levels of HMGB1 in serum of investigated patients in our study did not differ compared to the levels in controls. The findings thus suggest that HMGB1-positive MPO^+^MPs may be a better indicator of disease activity in AAV than HMGB1 levels detected in serum using ELISA assay.

Potential limitations of our study are the cross-sectional design, lack of disease controls, and relatively low number of patients in the investigated groups. Moreover, we did not observe any correlations between soluble markers measured by ELISA and MP expression. However, this discrepancy is somewhat expected as ELISA detects both soluble and bound proteins while flow cytometry only measures bound proteins as we have previously shown for HMGB1 and CD40 ligand [30]. Still, our study suggests that PTX3 and HMGB1 expressed on MPO^+^MPs might be used as promising biomarkers reflecting inflammation and disease activity in AAV patients. Our findings need to be replicated in a larger prospective study to validate these promising biomarkers of disease activity in patients with AAV.

## Electronic supplementary material

ESM 1 (DOCX 203 kb)

## Abbreviations

ANCA: Anti-neutrophil cytoplasmic antibody
AAV: ANCA-associated vasculitis
BVAS: Birmingham Vasculitis Activity Score
GPA: Granulomatosis with polyangiitis
HMGB1: High-mobility group box 1 protein
MPs: Microparticles
MPO: Myeloperoxidase
MPO^+^MPs: Myeloperoxidase-positive microparticles
MPA: Microscopic polyangiitis
NETs: Neutrophil extracellular traps
NMPs: Neutrophil-derived MPs
PPP: Platelet poor plasma
PR3: Proteinase 3
PTX3: Pentraxin-3
TWEAK: Tumor necrosis factor-like weak inducer of apoptosis
