# Supplementary material for: Microparticles expressing myeloperoxidase as potential biomarkers in anti-neutrophil cytoplasmic antibody (ANCA)-associated vasculitides (AAV)
Source: J Mol Med (Berl). 2020 Jul 30;98(9):1279–86. doi: 10.1007/s00109-020-01955-2 (PMC7447662; doi:10.1007/s00109-020-01955-2)

**Figure 1.** ROC curve for PTX3 serum concentrations, levels of MPO+ MPs expressing either PTX3 or HMGB1 in predicting disease activity in patients with AAV.

AUC (95% CI) are 0.8 (0.7-0.9), 0.7 (0.6 - 0.9) and 0.7 (0.5 - 0.9), respectively.

1. PTX3 in serum


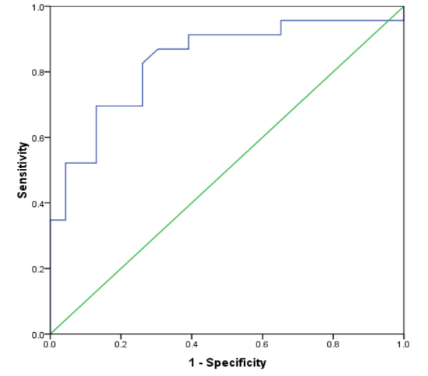


b) MPO+PTX3+ MPs c) MPO+HMGB1+ MPs


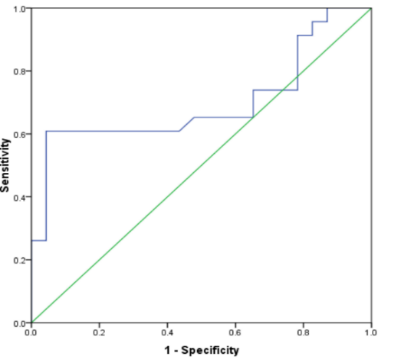

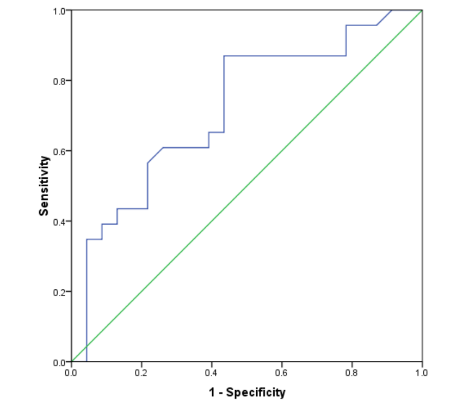

Supplement: Supplementary file 1 — (DOCX 203 kb) [file 109_2020_1955_MOESM1_ESM.docx]
